# Association between sodium–glucose cotransporter-2 (SGLT2) inhibitors and macular degeneration in patients with diabetes: a nationwide population-based study in Taiwan

**DOI:** 10.1007/s00592-024-02303-3

**Published:** 2024-05-24

**Authors:** Min-Yen Hsu, Kai-Shin Luo, Chien-Chih Chou, Yu-Hsiang Lin, Yu-Chien Hung, Wu-Lung Chuang, Stella Chin-Shaw Tsai, Heng-Jun Lin, Teng-Shun Yu, Fuu-Jen Tsai, Kuang-Hsi Chang

**Affiliations:** 1 School of Medicine, Chung Shan Medical University, Taichung City, 402 Taiwan; 2 Department of Ophthalmology, Chung Shan Medical University Hospital, Taichung City, 402 Taiwan; 3 Graduate Institute of Clinical Medicine, College of Medicine, National Taiwan University, Taipei, Taiwan; 4 School of Medicine, National Yang Ming Chiao Tung University, Taipei, Taiwan; 5 Department of Ophthalmology, Taichung Veterans General Hospital, Taichung, Taiwan; 6 Department of Post-Baccalaureate Medicine, College of Medicine, National Chung Hsing University, Taichung, Taiwan; 7 Division of Endocrinology and Metabolism, Department of Internal Medicine, Changhua Christian Hospital, Changhua City, 500 Taiwan; 8 Division of Endocrinology and Metabolism, Department of Internal Medicine, Lukang Christian Hospital, Changhua County, 505 Taiwan; 9 Department of Otolaryngology, Tungs’ Taichung MetroHarbor Hospital, Taichung, 435 Taiwan; 10 Rong Hsing Research Center for Translational Medicine, College of Life Sciences, National Chung Hsing University, Taichung, 402 Taiwan; 11 Management Office for Health Data, China Medical University Hospital, Taichung, 404 Taiwan; 12 College of Medicine, China Medical University, Taichung City, 404 Taiwan; 13 School of Chinese Medicine, College of Chinese Medicine, China Medical University, Taichung City, 404 Taiwan; 14 Department of Medical Research, China Medical University Hospital, China Medical University, Taichung City, 404 Taiwan; 15 Division of Medical Genetics, China Medical University Children’s Hospital, Taichung City, 404 Taiwan; 16 Department of Biotechnology and Bioinformatics, Asia University, Taichung City, 413 Taiwan; 17 Department of Medical Research, Tungs’ Taichung MetroHarbor Hospital, Taichung City, 435 Taiwan; 18 Center for General Education, China Medical University, Taichung City, 404 Taiwan; 19 General Education Center, Nursing and Management, Jen-Teh Junior College of Medicine, Miaoli County, 356 Taiwan

**Keywords:** Sodium-glucose cotransporter-2 (SGLT2), Macular degeneration (MD), National Health Insurance Research Database (NHIRD)

## Abstract

**Aims:**

Evidence showed that SGLT2 inhibitors have greater protective effects against retinal diseases compared to other hypoglycemic agents. Thus, we explore the association between SGLT2 inhibitor usage and macular degeneration (MD) in Taiwanese patients with diabetes.

**Methods:**

The National Health Insurance (NHI) program's claim data are released as the National Health Insurance Research Database (NHIRD). This database covers more than 99% of the residents in Taiwan. We included data on patients who were newly diagnosed with type 2 diabetes mellitus (ICD-9-CM: 250, exclude 250.1x; ICD-10-CM: E11), with an age at diagnosis of over 20 years as our study population. Patients who received (sodium-glucose cotransporter 2 inhibitor) SGLT2i (ATC code: A10BK) over 90 days in 2016–2019 were defined as the SGLT2i cohort. Conversely, patients who did never received SGLT2i were defined as the non-SGLT2i cohort. The exclusion criteria were having MD before the index date, receiving SGLT2i within 1–89 days, and missing data on sex, age, or days of SGLT2i usage. Two cohorts were matched by 1:1 propensity score matching, which was based on age, sex, payroll bracket grade, urbanization, comorbidities, and medications.

**Results:**

Compared to non-SGLT2i cohort, patients who received SGLT2i had a significantly lower risk of MD (adjusted hazard ratio = 0.70, 95%CI = 0.66–0.75).

**Conclusions:**

We found that SGLT2is has a strong protective effect against MD in patients with diabetes. SGLT2is may have benefits beyond glycemic control in patients with DR. However, additional clinical and experimental studies are required.

## Introduction

A novel type of drug, sodium-glucose cotransporter-2 (SGLT2) inhibitors, has been approved for diabetes treatment by the U.S. Food and Drug Administration (FDA) since 2014 [1]. SGLT2 is primarily expressed in the proximal convoluted tubule and functions as a reabsorption for glucose [2, 3]. SGLT2 inhibitors block the reabsorption of glucose by inhibiting SGLT2, thereby promoting glucose renal excretion; this lowers the elevated glucose levels of patients with diabetes and produces an osmotic diuretic effect [4].

Compared to other antihyperglycemic agents, SGLT2 inhibitors have a lower risk of hypoglycemia because they only affect the reabsorption of glucose that is filtered rather than blood glucose [5–7]. Moreover, SGLT2 inhibitors have cardiovascular protective effects and renal benefits [8–11]. In addition to cardiovascular and kidney problems, however, macular degeneration (MD) is also a complication among patients with diabetes [12–14]. A former review showed that the global incidence of MD was 1.59 (95% CrI 1.18–2.11) per 100 person-years [15]. A recent study revealed that from 2005 to 2014 [16], 3.75 to 3.95% of Taiwanese patients with diabetes experienced diabetic eye diseases, and 0.29 to 0.35% encountered poor vision and even blindness. A meta-analysis also showed that SGLT2 inhibitors have greater protective effects against retinal diseases compared to other hypoglycemic agents [17]. However, related evidence about SGLT2 inhibitor usage and MD is limited. Thus, we explore the association between SGLT2 inhibitor usage and MD in Taiwanese patients with diabetes.

## Methods

### Data source

The National Health Insurance (NHI) program of Taiwan was established in 1995, and this program's claim data are released as the National Health Insurance Research Database (NHIRD). This database covers more than 99% of the residents in Taiwan, and diseases such as type 2 diabetes mellitus or MD can be defined by the International Classification of Diseases, 9th Revision, Clinical Modification, and the International Classification of Diseases, 10th Revision (ICD-9-CM and ICD-10-CM). Additionally, therapeutic uses such as drugs for type 2 diabetes mellitus can be defined by the Anatomical Therapeutic Chemical (ATC) code. All analyses were performed in the Health and Welfare Data Center, and privacy policies were implemented to protect the privacy of beneficiaries. The Institutional Review Board (IRB) of China Medical University (CMUH110-REC3-133) has approved this study.

### Study population

We included data on patients who were newly diagnosed with type 2 diabetes mellitus (ICD-9-CM: 250, exclude 250.1x; ICD-10-CM: E11), with an age at diagnosis of over 20 years as our study population. Patients who received (sodium-glucose cotransporter 2 inhibitor) SGLT2i (ATC code: A10BK) over 90 days in 2016–2019 were defined as the SGLT2i cohort. Conversely, patients who did never received SGLT2i were defined as the non-SGLT2i cohort. The index date was defined as the first date of receiving SGLT2i in the SGLT2i cohort and as a random date between 2016 and 2019 in the non-SGLT2i cohort. The exclusion criteria were having MD before the index date, receiving SGLT2i within 1 to 89 days, and missing data on sex, age, or days of SGLT2i usage. Two cohorts were matched by 1:1 propensity score matching, which was based on age, sex, payroll bracket grade, urbanization, comorbidities, and medications.

### Main outcome and covariates

All study participants were followed from the index date until the onset of MD (ICD-9-CM: 362.5, ICD-10-CM: H35.3), withdrawal from the NHI program, or the end of 2019. The covariates included sex, age group (20–39, 40–59, 60 + years), payroll bracket grade (low, medium, high), urbanization (high, medium, low), comorbidities, and medications. Payroll bracket grade levels were defined as monthly income < 700 USD, 700–1300 USD, and > 1300 USD, respectively.

Comorbidities diagnosed before the index date, including hypertension (ICD-9-CM: 401–405; ICD-10-CM: I10-I13, I15, and N26.2), dyslipidemia (ICD-9-CM: 272; ICD-10-CM: E71.30, E75.21, E75.22, E75.24, E75.3, E75.5, E75.6, E77, E78.0-E78.6, E78.70, E78.79, E78.8, and E78.9), coronary artery disease (CAD) (ICD-9-CM: 410–414; ICD-10-CM: I20-I25), alcoholism (ICD-9-CM: 291, 303, 305.0, 571.0–571.3, 790.3, V11.3, and V79.1; ICD-10-CM: F10, K70, R78.0, and Z65.8), chronic obstructive pulmonary disease (COPD) (ICD-9-CM: 490–496, and 504–506; ICD-10-CM: J40-J47, and J64-J68), diabetic retinopathy (DR) (ICD-9-CM: 362.0; ICD-10-CM: E11.31-E11.35), and macular edema (ICD-9-CM: 362.83; ICD-10-CM: H35.81), were considered.

Eight common antidiabetic drugs, including metformin, sulphonylurea, meglitinides, a-glucosidase inhibitors (AGI), thiazolidinedione, dipeptidyl peptidase 4 inhibitors (DPP4i), glucagon-like peptide-1 receptor agonists (GLP-1RA), and insulin, were considered.

### Statistical method

Mean and standard error (SD) were used to perform characteristics of continuous variables such as age and follow-up time, and percentage was used to perform characteristics of category variables. The difference can be evaluated using the standardized mean difference (SMD). The incidence rate was performed per 1,000 person-years (PY) and was calculated by n/PY*1,000. Univariable and multivariable Cox regression models were used to calculate the hazard ratio and the 95% interval estimation of the hazard ratio. The multivariable Cox regression adjusted age, sex, payroll bracket grade, urbanization, comorbidities, and medications. All analyses were performed using SAS version 9.4 (SAS Institute, Inc., Cary, NC, USA), and *p* values of 0.05 or less were considered statistically significant.

## Result

Table 1 presents the demographic characteristics of the two cohorts. There were 147,664 patients matched 1:1 with another 147,664 patients in both cohorts. The SMD shows that age, sex, payroll bracket grade, urbanization, comorbidities, and medications had no significant difference between non-SGLT2i and SGLT2i cohorts. There were approximately 58% male patients among patients with type 2 diabetes mellitus (DM). The major urbanization level was high, with approximately 52%, and the major payroll bracket grade level was medium, with approximately 54%. The top two comorbidities, including hypertension and dyslipidemia, were performed. The proportions of patients receiving medications with and without SGLT2i were as follows: metformin (97.34% versus 97.51%), sulphonylurea (83.63% versus 84.25%), meglitinides (18.06% versus 18.12%), AGI (36.71% versus 35.82%), thiazolidinedione (39.21% versus 37.94%), DPP4i (72.00% versus 71.69%), GLP-1RA (2.02% versus 1.89%), and insulin (40.60% versus 41.55%), respectively. The mean (SD) follow-up times were 2.24 (0.78) years in the study group and 2.21 (0.98) years in the control group.

**Table 1:** Comparison of demographic characteristics and comorbidities between T2DM patients with SGLT2i and controls

Variables	Groups	Non-SGLT2i (N = 147,664)		SGLT2i (N = 147,664)		SMD
Sex	Male	85,938	58.20	85,689	58.03	0.003
Age	20–39	9762	6.61	9843	6.67	0.002
	40–59	64,890	43.94	67,161	45.48	0.031
	60 +	73,012	49.44	70,660	47.85	0.032
	Mean, (SD)	58.55	11.88	58.10	11.71	0.038
Payroll bracket grade	Low	31,923	21.62	30,221	20.47	0.028
	Medium	79,127	53.59	79,441	53.80	0.004
	High	36,614	24.80	38,002	25.74	0.022
Urbanization	Low	12,460	8.44	12,625	8.55	0.004
	Medium	57,819	39.16	57,729	39.09	0.001
	High	77,385	52.41	77,310	52.36	0.001
Comorbidities	Hypertension	112,062	75.89	110,259	74.67	0.028
	Dyslipidemia	123,897	83.90	123,867	83.88	0.001
	CAD	45,307	30.68	44,244	29.96	0.016
	Alcoholism	3571	2.42	3804	2.58	0.010
	COPD	37,916	25.68	37,626	25.48	0.005
	DR	22,339	15.13	22,248	15.07	0.002
	Macular edema	1127	0.76	786	0.53	0.029
Medication	Metformin	143,986	97.51	143,738	97.34	0.011
	Sulphonylurea	124,413	84.25	123,498	83.63	0.017
	Meglitinides	26,761	18.12	26,661	18.06	0.002
	AGI	52,895	35.82	54,202	36.71	0.018
	Thiazolidinedione	56,030	37.94	57,897	39.21	0.026
	DPP4i	105,865	71.69	106,320	72.00	0.007
	GLP-1RA	2788	1.89	2976	2.02	0.009
	Insulin	61,359	41.55	59,956	40.60	0.019
Follow-up time	Mean, (SD)	2.21	0.98	2.24	0.78	0.029

SMD, standardized mean difference; CAD, coronary artery disease; COPD, chronic obstructive pulmonary disease; DR, diabetic retinopathy; payroll bracket grade, low: < 700 USD, medium: 700–1300 USD, high: > 1300 USD

Table 2 shows the risk of MD according to whether the patients have SGLT2i, comorbidities, or medications. Compared to patients who did not receive SGLT2i, patients who received SGLT2i had a significantly lower risk of MD (adjusted hazard ratio [aHR] = 0.70, 95% CI = 0.66–0.75). For different age groups, we found that patients over 60 had a higher risk of MD compared to those aged 20–39 (aHR = 4.06, 95% CI = 3.17–5.18). There was no significantly different risk of MD between female and male patients. Patients with a payroll bracket grade higher than 700 USD had lower risks of MD than those with a payroll bracket grade lower than 700 USD. Moreover, patients with comorbidities, including CAD (aHR = 1.11, 95% CI = 1.03–1.19), DR (aHR = 2.47, 95% CI = 2.30–2.65), macular edema (aHR = 1.65, 95% CI = 1.32–2.05), as well as those who received medications such as AGI (aHR = 1.16, 95% CI = 1.08–1.24), thiazolidinedione (aHR = 1.09, 95% CI = 1.01–1.16), DPP4i (aHR = 1.12, 95% CI = 1.02–1.22), and insulin (aHR = 1.13, 95% CI = 1.06–1.21) had higher risks of MD compared with the corresponding groups.

**Table 2:** Hazard ratios and 95% confidence intervals of macular degeneration development

Variables	Groups	n	PY	IR	cHR (95% CI)	aHR (95% CI)
SGLT2i	No	2185	326,735	6.69	1.00	1.00
	Yes	1536	330,514	4.65	0.69 (0.65, 0.74)	0.70 (0.66, 0.75)
Sex	Female	1772	280,113	6.33	1.00	1.00
	Male	1949	377,136	5.17	0.82 (0.77, 0.87)	0.98 (0.92, 1.04)
Age	20–39	68	43,545	1.56	1.00	1.00
	40–59	954	296,118	3.22	2.06 (1.61, 2.64)	1.85 (1.44, 2.37)
	60 +	2699	317,587	8.50	5.45 (4.28, 6.93)	4.06 (3.17, 5.18)
Payroll bracket grade	Low	935	137,373	6.81	1.00	1.00
	Medium	1962	353,283	5.55	0.82 (0.75, 0.88)	0.88 (0.82, 0.96)
	High	824	166,593	4.95	0.73 (0.66, 0.80)	0.90 (0.81, 0.98)
Urbanization	Low	335	56,000	5.98	1.00	1.00
	Medium	1442	257,064	5.61	0.94 (0.83, 1.06)	0.95 (0.84, 1.07)
	High	1944	344,185	5.65	0.94 (0.84, 1.06)	0.98 (0.87, 1.10)
*Comorbidities*						
Hypertension	No	683	164,022	4.16	1.00	1.00
	Yes	3038	493,227	6.16	1.48 (1.36, 1.61)	1.00 (0.91, 1.09)
Dyslipidemia	No	575	105,464	5.45	1.00	1.00
	Yes	3146	551,785	5.70	1.04 (0.96, 1.14)	0.92 (0.84, 1.01)
CAD	No	2299	461,302	4.98	1.00	1.00
	Yes	1422	195,947	7.26	1.46 (1.37, 1.56)	1.11 (1.03, 1.19)
Alcoholism	No	3665	641,466	5.71	1.00	1.00
	Yes	56	15,783	3.55	0.62 (0.48, 0.81)	0.78 (0.60, 1.02)
COPD	No	2624	491,705	5.34	1.00	1.00
	Yes	1097	165,544	6.63	1.24 (1.16, 1.33)	1.03 (0.95, 1.10)
DR	No	2361	556,642	4.24	1.00	1.00
	Yes	1360	100,607	13.52	3.19 (2.98, 3.41)	2.47 (2.30, 2.65)
Macular edema	No	3637	653,015	5.57	1.00	
	Yes	84	4235	19.84	3.57 (2.87, 4.43)	1.65 (1.32, 2.05)
*Medication*						
Metformin	No	61	15,422	3.96	1.00	1.00
	Yes	3660	641,827	5.70	1.43 (1.11, 1.84)	0.96 (0.74, 1.25)
Sulphonylurea	No	378	99,082	3.82	1.00	1.00
	Yes	3343	558,167	5.99	1.56 (1.41, 1.74)	1.02 (0.91, 1.14)
Meglitinides	No	2845	538,228	5.29	1.00	1.00
	Yes	876	119,021	7.36	1.39 (1.29, 1.50)	1.01 (0.93, 1.09)
AGI	No	1939	415,606	4.67	1.00	1.00
	Yes	1782	241,644	7.37	1.58 (1.48, 1.68)	1.16 (1.08, 1.24)
Thiazolidinedione	No	1861	399,786	4.65	1.00	1.00
	Yes	1860	257,463	7.22	1.55 (1.45, 1.65)	1.09 (1.01, 1.16)
DPP4i	No	730	179,576	4.07	1.00	1.00
	Yes	2991	477,673	6.26	1.54 (1.42, 1.67)	1.12 (1.02, 1.22)
GLP-1RA	No	3646	645,874	5.65	1.00	1.00
	Yes	75	11,375	6.59	1.18 (0.94, 1.48)	1.11 (0.88, 1.39)
Insulin	No	1810	388,269	4.66	1.00	1.00
	Yes	1911	268,980	7.10	1.52 (1.43, 1.63)	1.13 (1.06, 1.21)

n, number of patients with macular degeneration; IR, incidence rate (per 1000 person-years); PY, person-year; cHR, crude hazard ratio; aHR, adjusted hazard ratio; CAD, coronary artery disease; COPD, chronic obstructive pulmonary disease; DR, diabetic retinopathy; AGI, a-glucosidase inhibitors; payroll bracket grade, low: < 700 USD, medium: 700–1300 USD, high: > 1300 USD

When patients were categorized according to covariates, the aHRs were statistically lower in the SGLT2i group than in the control group, except in alcoholism patients, macular edema patients, without metformin, without sulphonylurea, and with GLP-1RA; the aHR were 0.72 (95% CI = 0.43–1.23), 0.78 (95% CI = 0.50–1.24), 0.71 (95% CI =  0.42–1.18), 0.82 (95% CI = 0.67–1.00), and 0.63 (95% CI = 0.40, 1.01), respectively (Table 3).

**Table 3:** Hazard ratios and 95% confidence intervals of macular degeneration development with and without SGLT2i stratified by age, comorbidities, and medication

Variables	Groups	Non-SGLT2i			SGLT2i			cHR (95%CI)	aHR (95%CI)
		n	PY	IR	n	PY	IR		
Sex	Female	1020	139,243	7.33	752	140,869	5.34	0.72 (0.66, 0.79)	0.74 (0.67, 0.82)
	Male	1165	187,492	6.21	784	189,645	4.13	0.66 (0.60, 0.72)	0.66 (0.61, 0.73)
Age	20–39	46	21,389	2.15	22	22,156	0.99	0.45 (0.27, 0.75)	0.47 (0.28, 0.78)
	40–59	565	144,789	3.90	389	151,329	2.57	0.66 (0.58, 0.75)	0.65 (0.57, 0.74)
	60 +	1574	160,557	9.80	1125	157,030	7.16	0.72 (0.67, 0.78)	0.72 (0.67, 0.78)
Payroll bracket grade	Low	515	69,631	7.40	420	67,742	6.20	0.82 (0.72, 0.94)	0.81 (0.71, 0.93)
	Medium	1182	175,537	6.73	780	177,746	4.39	0.64 (0.59, 0.71)	0.66 (0.60, 0.72)
	High	488	81,567	5.98	336	85,026	3.95	0.66 (0.58, 0.76)	0.69 (0.60, 0.79)
Urbanization	Low	219	27,974	7.83	116	28,026	4.14	0.53 (0.42, 0.66)	0.56 (0.45, 0.70)
	Medium	849	127,773	6.64	593	129,291	4.59	0.68 (0.61, 0.76)	0.69 (0.62, 0.77)
	High	1117	170,988	6.53	827	173,197	4.77	0.73 (0.66, 0.80)	0.73 (0.67, 0.80)
*Comorbidities*									
Hypertension	No	381	79,820	4.77	302	84,202	3.59	0.75 (0.64, 0.87)	0.69 (0.59, 0.80)
	Yes	1804	246,915	7.31	1234	246,312	5.01	0.68 (0.63, 0.73)	0.70 (0.65, 0.75)
Dyslipidemia	No	314	51,898	6.05	261	53,566	4.87	0.80 (0.67, 0.94)	0.77 (0.65, 0.90)
	Yes	1871	274,837	6.81	1275	276,948	4.60	0.67 (0.63, 0.72)	0.69 (0.64, 0.74)
CAD	No	1326	228,547	5.80	973	232,755	4.18	0.72 (0.66, 0.78)	0.72 (0.66, 0.78)
	Yes	859	98,188	8.75	563	97,759	5.76	0.65 (0.58, 0.72)	0.67 (0.60, 0.74)
Alcoholism	No	2156	319,265	6.75	1509	322,201	4.68	0.69 (0.64, 0.73)	0.70 (0.65, 0.75)
	Yes	29	7470	3.88	27	8313	3.25	0.82 (0.49, 1.39)	0.72 (0.43, 1.23)
COPD	No	1531	244,421	6.26	1093	247,285	4.42	0.70 (0.65, 0.76)	0.71 (0.66, 0.77)
	Yes	654	82,314	7.95	443	83,229	5.32	0.66 (0.58, 0.74)	0.67 (0.60, 0.76)
DR	No	1398	277,059	5.05	963	279,583	3.44	0.68 (0.62, 0.74)	0.68 (0.63, 0.74)
	Yes	787	49,676	15.84	573	50,931	11.25	0.70 (0.63, 0.78)	0.72 (0.65, 0.80)
Macular edema	No	2133	324,290	6.58	1504	328,724	4.58	0.69 (0.65, 0.74)	0.70 (0.65, 0.74)
	Yes	52	2445	21.27	32	1790	17.88	0.84 (0.54, 1.30)	0.78 (0.50, 1.24)
*Medication*									
Metformin	No	32	7412	4.32	29	8010	3.62	0.83 (0.50, 1.37)	0.71 (0.42, 1.18)
	Yes	2153	319,323	6.74	1507	322,504	4.67	0.69 (0.64, 0.73)	0.70 (0.65, 0.75)
Sulphonylurea	No	192	48,373	3.97	186	50,709	3.67	0.91 (0.75, 1.12)	0.82 (0.67, 1.00)
	Yes	1993	278,362	7.16	1350	279,805	4.82	0.67 (0.62, 0.72)	0.68 (0.64, 0.73)
Meglitinides	No	1664	268,391	6.20	1181	269,837	4.38	0.70 (0.65, 0.75)	0.69 (0.64, 0.74)
	Yes	521	58,344	8.93	355	60,677	5.85	0.65 (0.57, 0.74)	0.73 (0.63, 0.83)
AGI	No	1128	209,166	5.39	811	206,439	3.93	0.72 (0.66, 0.79)	0.72 (0.66, 0.79)
	Yes	1057	117,569	8.99	725	124,075	5.84	0.64 (0.59, 0.71)	0.68 (0.61, 0.74)
Thiazolidinedione	No	1086	202,087	5.37	775	197,699	3.92	0.72 (0.66, 0.79)	0.72 (0.66, 0.79)
	Yes	1099	124,648	8.82	761	132,816	5.73	0.64 (0.59, 0.71)	0.68 (0.62, 0.74)
DPP4i	No	432	91,202	4.74	298	88,374	3.37	0.70 (0.61, 0.82)	0.67 (0.57, 0.77)
	Yes	1753	235,533	7.44	1238	242,141	5.11	0.68 (0.63, 0.73)	0.71 (0.66, 0.76)
GLP-1RA	No	2144	321,941	6.66	1502	323,933	4.64	0.69 (0.65, 0.74)	0.70 (0.66, 0.75)
	Yes	41	4794	8.55	34	6581	5.17	0.58 (0.37, 0.92)	0.63 (0.40, 1.01)
Insulin	No	1086	194,081	5.60	724	194,188	3.73	0.66 (0.60, 0.73)	0.65 (0.59, 0.72)
	Yes	1099	132,654	8.28	812	136,326	5.96	0.71 (0.65, 0.78)	0.74 (0.67, 0.81)

n, number of patients with macular degeneration; IR, incidence rate (per 1000 person-years); PY, person-year; cHR, crude hazard ratio; aHR, adjusted hazard ratio; CAD, coronary artery disease; COPD, chronic obstructive pulmonary disease; DR, diabetic retinopathy; payroll bracket grade, low: < 700 USD, medium: 700–1300 USD, high: > 1300 USD

### Discussions

The study investigates the use of SGLT2 inhibitors. SGLT2 inhibitors block the reabsorption of glucose by inhibiting SGLT2, promoting glucose renal excretion, lowering the elevated glucose levels of patients with diabetes, and producing an osmotic diuretic effect [18–20]. This study explored the association between SGLT2 inhibitors in patients with DM and the development of MD. The study used data from the Longitudinal Generation Tracking Database (LGTD), which was derived from a large database of a single-payer NHI program initiated in Taiwan since 1995, called the NHIRD. We aimed to explore the risk of MD in patients with diabetes treated with SGLT2 inhibitors. We found that the incidence of MD was lower in diabetic patients treated with SGLT2 inhibitors than in those treated without SGLT2 inhibitors. This may imply some positive correlation between SGLT2 inhibitors and the prevention of MD.

The mechanisms underlying the protective effect of SGLT2 inhibitors that reduce the risk of MD remain understudied. During the early stage of diabetic retinopathy, pericyte swelling and pericyte loss occur, which results in microaneurysm formation in the retina. The swollen pericytes lose their contractile ability and lead to retinal hyperperfusion [21, 22]. These processes result in a similar clinical observation as the early MD disease sign, edematous macula. The SGLT2 inhibitor has been proven to decrease inflammatory responses and oxidative processes, such as changing the phenotype of astrocytes and microglia into pro-inflammatory ones [22], which also plays a crucial role in MD disease. SGLT2 inhibitors elevate the ratios of adenosine diphosphate/adenosine triphosphate (ATP) and AMP/ATP, leading to a decrease in ATP production and oxidative phosphorylation. Consequently, cellular ATP levels are reduced, which triggers AMPK activation. AMPK activation hinders protein synthesis and cell proliferation. A previous study has shown that metformin exerts its anti-inflammatory and antioxidative effects primarily through the activation of AMP-activated protein kinase (AMPK). This activation leads to the inhibition of nuclear factor-kappa B, a reduction in reactive oxygen species, and the inhibition of the mammalian target of rapamycin. AMPK, a metabolic-sensing Ser/Thr kinase expressed in all cell types, plays a central role in maintaining energy homeostasis and regulating metabolic stress. It also contributes to the dysfunction of the metabolic ecosystem, potentially influencing the pathogenesis of age-related macular degeneration (AMD).

Metformin's protective effects against oxidative damage and mitochondrial dysfunction are considered additional mechanisms for its potential efficacy in AMD treatment [23]. These effects have been confirmed in retinal pigment epithelial (RPE) cells, where metformin stimulates autophagy through the activation of the AMPK pathway, effectively shielding RPE cells from oxidative damage. Furthermore, metformin safeguards photoreceptors and RPE cells from acute injury and delays inherited retinal degeneration by reducing oxidative stress and increasing mitochondrial energy production. Additionally, metformin demonstrates anti-inflammatory properties on the RPE by attenuating pro-inflammatory and adhesion molecule genes. This is particularly relevant in early AMD, characterized by drusen formation, as it results from an inflammatory reaction triggered by RPE damage. Thus, the anti-inflammatory and antioxidant effects of metformin help protect the RPE from early AMD lesions [13, 24]. Thus, in our study, SGLT2 inhibitors showed a similar effect to metformin and resulted in a decreased relative risk of MD in patients with DM.

Subretinal fibrosis and geographic atrophy are challenging clinical situations. Type IV collagen synthesis is increased under high-glucose conditions in bovine pericytes. SGLT2 inhibitors may inhibit mesangial expansion in the glomerulus and microvessel occlusion in the retina (22). Thus, the collagen synthesis modulation could be a positive impact on MD disease prevention.

The study has several strengths, such as the use of a large database, adjustment of potential confounders, and multivariate Cox proportional hazards models. However, the study has some limitations: It is a retrospective cohort study that could not determine the causal relationship between SGLT2 inhibitors and MD. Moreover, the study was conducted in Taiwan; therefore, the results may not be generalizable to other populations. Finally, the Image evaluation and results of hematology test are not available from NHIRD. However, all the patients who need anti-VEFG drugs to treat macular degeneration disease will turn in optical coherence tomography (OCT) image for audited. Similarly, all the patients who need anti-VEFG drugs to treating diabetic macular edema will turn in HbA1C data for audited. The data of HbA1C for first diagnosis should below 10%. Overall, the study suggests that SGLT2 inhibitors may have a protective effect on MD in patients with diabetes.

In conclusion, we found that SGLT2is has a stronger protective effect against MD in patients with diabetes than diuretics. SGLT2is may have benefits beyond glycemic control in patients with DR. Compared with other anti-diabetic drugs, SGLT2 inhibitors show strong ability of anti-inflammatory and antioxidant effects by regulating adenosine diphosphate/adenosine triphosphate (ATP) and AMP/ATP pathway. SGLT2 inhibitor may have a better chance to propone macular degeneration disease process and lead to the protecting effect. However, additional clinical and experimental studies are required.

## Data Availability

Information is accessible through the National Health Insurance Research Database (NHIRD) provided by the Taiwan National Health Insurance (NHI) Administration. However, due to legal constraints stipulated by the Taiwan government under the "Personal Information Protection Act," the data cannot be released to the public. If you wish to obtain the data, you may submit formal requests to the NHIRD via their website (https://dep.mohw.gov.tw/DOS/lp-2506-113.html).
